# Global Gene Responses of Resistant and Susceptible Sugarcane Cultivars to *Acidovorax avenae* subsp. *avenae* Identified Using Comparative Transcriptome Analysis

**DOI:** 10.3390/microorganisms8010010

**Published:** 2019-12-19

**Authors:** Na Chu, Jing-Ru Zhou, Hua-Ying Fu, Mei-Ting Huang, Hui-Li Zhang, San-Ji Gao

**Affiliations:** National Engineering Research Center for Sugarcane, Fujian Agriculture and Forestry University, Fuzhou 350002, Fujian, China; 13850139095@163.com (N.C.); zjr18635465333@163.com (J.-R.Z.); mddzyfhy@163.com (H.-Y.F.); hmt159379@163.com (M.-T.H.); huilizhang2014@163.com (H.-L.Z.)

**Keywords:** *Saccharum* spp., red stripe disease, *Acidovorax avenae* subsp. *avenae*, transcriptome, disease resistance

## Abstract

Red stripe disease in sugarcane caused by *Acidovorax avenae* subsp. *avenae* (*Aaa*) is related to serious global losses in yield. However, the underlying molecular mechanisms associated with responses of sugarcane plants to infection by this pathogen remain largely unknown. Here, we used Illumina RNA-sequencing (RNA-seq) to perform large-scale transcriptome sequencing of two sugarcane cultivars to contrast gene expression patterns of plants between *Aaa* and mock inoculations, and identify key genes and pathways involved in sugarcane defense responses to *Aaa* infection. At 0–72 hours post-inoculation (hpi) of the red stripe disease-resistant cultivar ROC22, a total of 18,689 genes were differentially expressed between *Aaa*-inoculated and mock-inoculated samples. Of these, 8498 and 10,196 genes were up- and downregulated, respectively. In MT11-610, which is susceptible to red stripe disease, 15,782 genes were differentially expressed between *Aaa*-inoculated and mock-inoculated samples and 8807 and 6984 genes were up- and downregulated, respectively. The genes that were differentially expressed following *Aaa* inoculation were mainly involved in photosynthesis and carbon metabolism, phenylpropanoid biosynthesis, plant hormone signal transduction, and plant–pathogen interaction pathways. Further, qRT-PCR and RNA-seq used for additional validation of 12 differentially expressed genes (DEGs) showed that eight genes in particular were highly expressed in ROC22. These eight genes participated in the biosynthesis of lignin and coumarin, as well as signal transduction by salicylic acid, jasmonic acid, ethylene, and mitogen-activated protein kinase (MAPK), suggesting that they play essential roles in sugarcane resistance to *Aaa*. Collectively, our results characterized the sugarcane transcriptome during early infection with *Aaa*, thereby providing insights into the molecular mechanisms responsible for bacterial tolerance.

## 1. Introduction

Sugarcane accounts for 80% of sugar production worldwide, and also provides cellulosic biomass for grazing livestock and biofuel production [1]. Modern sugarcane cultivars (2n = 100–130) originate from the nobilization process, which involves interspecific hybridization between *Saccharum officinarum* (also known as noble cane, 2n = 80) and *S. spontaneum* (2n = 40–128) and further backcrossing to *S. officinarum* [2]. The high sugar content of these hybrids originates from *S. officinarum* whereas other traits such as hardiness, disease resistance, and ratooning can be attributed to *S. spontaneum* [3,4]. Modern sugarcane varieties have a narrow genetic base due to their common origins from some cultivars that were produced in the early 1900s, and this lack of genetic diversity has hindered attempts to increase sugarcane production and resistance to diverse abiotic and biotic stresses [5,6]. As a vegetatively propagated crop, sugarcane has become increasingly vulnerable to certain viral pathogens and other non-fungal pathogens [7], resulting in varietal degeneration in sugarcane, as evidenced by a loss of vigor in sugarcane cultivars [8]. Yield losses in sugarcane due to biotic stresses can be up to 10–15% worldwide [9].

In addition to the three main bacterial diseases of sugarcane, namely ratoon stunting caused by *Leifsonia xyli* subsp. xyli (*Lxx*), leaf scald caused by *Xanthomonas albilineans*, and gumming caused by *X. vasicola* pv. *vasculorum* [10], red stripe disease is another important bacterial disease caused by the bacterium *Acidovorax avenae* subsp. *avenae* (*Aaa*) [11]. Red stripe disease is present in more than 50 countries, including China [11,12]. The discovery of this disease and its causal agents were tracked to the 1920s in Hawaii [13]. The causal agent of red stripe disease was formerly referred to as *Pseudomonas rubrilineans* [13] but was later updated to *Aaa* under the genus *Acidovorax* of the family Comamonadaceae [14]. In recent years, *Aaa* was also successfully isolated from diseased sugarcane plants in Argentina [15] and China [11,16]. Two types of disease symptoms, leaf stripe and top rot, appear or co-exist in sugarcane affected by *Aaa*. The leaf stripe symptoms manifest as uniformly long and narrow dark-red stripes across the longitudinal axis of the leaf whereas top rot is associated with rotting at the top of the plant [13]. An outbreak of red stripe disease recently occurred in Argentina and caused a 30% loss in yield [15]. In China, sugarcane red stripe disease has been found in many provinces since the 1980s and recent field surveys have revealed that this disease has an incidence of up to 80% in highly susceptible sugarcane cultivars [11,16]. Thus, strategies for the biological prevention and increased resistance of sugarcane are urgently needed. However, we have limited insight into the molecular and pathogenic mechanisms of *Aaa* in sugarcane.

Next generation high-throughput sequencing (RNA sequencing) has been widely used for transcriptome studies of plants under biotic and abiotic stresses, and have helped to increase our understanding of the underlying genes and gene regulatory networks mediated by different stresses [1,17]. This RNA sequencing (RNA-seq) technology has been applied to examine how sugarcane responds to diverse pathogens infections, such as *Sorghum mosaic virus* (SrMV) and *Sugarcane streak mosaic virus* (SCSMV) associated with mosaic disease [18,19], *Sporisorium scitamineum* that causes smut [20,21], *Fusarium verticillioides* that causes pokkah boeng [22], and three main pathogenic bacteria, *Lxx* [23,24], *X. albilineans* [25], and *Aaa* [26]. These studies provided a basis for understanding the molecular mechanisms of sugarcane–pathogen interactions, but sugarcane plants have an array of different defense mechanisms against various invading pathogens.

Until now, only one investigation of sugarcane responses to *Aaa* infection has been conducted. This study used transcriptome analysis of limited samples, namely, two biological replicates of mock- and *Aaa*-infected plants at one time point [26]. Identification of additional differentially expressed genes (DEGs) and related defense pathways that are triggered in response to *Aaa* infection of sugarcane is needed. In this study, we used Illumina RNA-seq technology to carry out a comparative transcriptome analysis of two resistant and susceptible sugarcane cultivars inoculated with *Aaa* at four time points. Our results provide new insights for understanding the molecular defense mechanisms involved in sugarcane response to *Aaa* infection.

## 2. Materials and Methods

### 2.1. Plant Growth, Bacteria Inoculation and Leaf Tissue Sampling

Two sugarcane cultivars, ROC22 (resistant to red stipe) and MT11-610 (susceptible to red stipe), supplied by the Taiwan Sugar Corporation (Taiwan, China) and Fujian Academy of Agricultural Sciences (Zhangzhou, China), respectively, were used. Buds from the two cultivars were germinated and cultured in a growth chamber at 28 °C and 60% humidity under 16/8 h light/dark photoperiod until the plants were approximately 15–20 cm tall (3–5 leaf stage). Eighteen plants of each cultivar were injected with a bacterial suspension (10^8^ CFU/mL) of *Aaa* SC-026 following the protocol described by Li et al. [11]. Another ten plants from each cultivar were injected with only sterile selective nutrient broth (NB) medium (10.0 g/L peptone, 3.0 g/L beef extract, and 5.0 g/L sodium chloride) and used as controls. Leaf samples from the two cultivars were collected for RNA-seq at four time points: 0 h (R0_CK and S0_CK), 24 h (R24_Aaa and S24_Aaa), 48 h (R48_Aaa and S48_Aaa), and 72 h (R72_Aaa and S72_Aaa) post-inoculation (hpi). At each sampling time point, inoculated leaf tissues from six plants (three replicates) of each treatment were sampled and immediately snap-frozen in liquid nitrogen and stored at −80 °C until RNA and DNA extraction. A total of 24 samples were used for Illumina RNA-seq deep sequencing and were also used for the quantitative detection of bacterial populations in each sample.

### 2.2. RNA-Sequencing and de novo Transcriptome Assembly

After the quality and quantity of extracted total leaf RNA was determined, 1.5 μg RNA from each sample was used as input material for RNA-seq. The library preparations were sequenced on an Illumina NovaSeq 6000 platform at Novogene Bioinformatics Institute (Beijing, China) and 150-bp paired-end reads (PE 150) were generated. Prior to generating the clean reads, the raw reads were used to remove reads containing adapters, poly-N, and low-quality reads. Meanwhile, the Q20, Q30, GC-content and sequence duplication level of the clean reads were calculated. These high clean reads were used for de novo transcriptome assembly by Trinity (v2012-10-05) [27] with the min_kmer_cov set to 2 by default. Default values were used for all other parameters. After transcriptome assembly, each unigene was annotated using the nucleotide database Nt (NCBI nucleotide sequences) and the protein databases Nr (NCBI non-redundant protein sequences), Pfam (Protein family) and Swiss-Prot (a manually annotated and reviewed protein sequence database), and assigned to functional categories in the KOG/COG (clusters of orthologous groups of proteins/eukaryotic ortholog groups), GO (gene ontology) and KEGG (Kyoto encyclopedia of genes and genomes) databases using BLASTx with an E value cutoff of 10^−5^. The Illumina sequencing data for sugarcane infected with *Aaa* were deposited in the NCBI Short Read Archive (SRA) database as BioProject accession number PRJNA579959.

### 2.3. Differential Expression Analysis

Gene expression levels were estimated by RSEM for each sample using default parameters [28]. Briefly, clean data were mapped back onto the assembled transcriptome and the read count for each gene was then obtained from the mapping results. The abundance of each assembled transcript was evaluated using fragments per kilobase per million reads (FPKM) [29]. For genes with more than one alternative transcript, the longest transcript was selected for FPKM calculation. Differential expression analysis between sample pairs was performed using the DESeq2 package [30]. The parameters of |log_2_(fold change)|>1.5 and *p*-value < 0.005 were set as the threshold for significantly differential expression. GO enrichment analysis of DEGs was conducted using the GOseq R package Wallenius non-central hyper-geometric distribution [31]. GO terms demonstrating significant enrichment were those with a *p*-value < 0.005. KEGG enrichment analysis of DEGs was carried out using KOBAS 2.0 with *p*-value < 0.05 [32,33].

### 2.4. Real-Time Quantitative PCR Assay

To validate the credibility of DEGs screened by RNA-Seq, 12 candidate genes comprising 9 and 3 up- and downregulated genes, respectively, were validated by quantitative real-time PCR (qRT-PCR). Based on the 12 DEGs sequences from the cDNA library, specific primers were designed for qRT-PCR assays using primer premier 5.0 software (Table S1). The glyceraldehyde-3-phosphate dehydrogenase (*GAPDH*) was used as an internal control. The first-strand cDNA was synthesized with a First Strand cDNA Synthesis Kit (Takara, Dalian, China) with the templates for 24 RNA samples that were from the above-mentioned RNA-seq experiments. Further, qPCR assays were carried out using SYBR Premix Ex TaqII (Takara, Dalian, China) on a QuantStudio^®^ Real-Time PCR system (Applied Biosystems, Foster City, USA). The reactions included 10 μL TB GreenTM Premix EX Taq II, 1 μL cDNA, 0.4 μL forward primer (10 mM), 0.4 μL reverse primer (10 mM), 0.4 μL ROX Reference Dye II and 7.8 μL nuclease-free water. The qPCR reactions involved denaturation at 95 °C for 30 s, followed by 40 cycles of 5 s at 95 °C, and 34 s at 60 °C. The qRT-PCR data were analyzed using the 2^−ΔΔCt^ quantitative method to determine differences in gene expression [34]. Three biological replicates and three technological replicates were used for each sample.

## 3. Results

### 3.1. Transcriptome Sequencing and Assembly

To globally investigate genes involved in sugarcane response to *Aaa* infection, 24 cDNA libraries were sequenced (Table S2). Between 55,408,650 and 62,218,354 raw reads were obtained for the *Aaa* susceptible ROC22 cultivar and between 58,224,389 and 65,758,625 raw reads were obtained for the *Aaa* resistant MT11-610 cultivar. These raw reads were filtered to yield 59,959,395, 61,259,067, 54,282,803, 57,434,943, 57,284,497, 64,485,665, 57,875,395, and 61,269,024 clean reads for R0_CK, R24_Aaa, R48_Aaa, R72_Aaa, S0_CK, S24_Aaa, S48_Aaa, and S72_Aaa, respectively. The GC content, Q30 and mapping percentage of the eight libraries was more than 55%, 90%, and 65%, respectively. The de novo sequence assembly of transcripts from RNA-seq reads showed that 579,561 transcripts were generated with an average length of 789 bp and an N50 of 1144 bp, whereas 507,558 unigenes were obtained with an average length of 860 bp and an N50 of 1200 bp (Table 1). The transcript and unigene lengths were between 201 bp and 15,496 bp. The length distribution of nucleotides ranged from 200 to 500 bp, accounting for 48.0% and 41.2% of the total transcripts and unigenes, respectively (Table 1).

### 3.2. Gene Annotation of Assembled Unigenes

All of the assembled high-quality unigenes were annotated by searching against seven public databases. Significant annotation matches were found for 264,027 (52.0%) in the Nr database, 352,564 (69.5%) in the Nt database, 149,669 (29.5%) in the Swiss-Prot database, 199,556 (39.3%) in the GO database and 173,258 (34.1%) in the Pfam database. Overall, 394,186 (77.7%) unigenes were successfully annotated in at least one of the seven databases, whereas 25,350 (5.0%) unigenes appeared in all seven databases (Figure S1A). Most of the annotated sequences corresponding to known nucleotide sequences of plant species in the NR database were matched with *Sorghum bicolor* (49.5%), *Zea mays* (24.8%), other species (10.0%), *Setaria italic* (9.7%), *Oryza sativa* (3.6%), and *Saccharum* hybrids (2.4%) (Figure S1B).

### 3.3. GO and KEGG Functional Classification of Unigenes

GO analysis revealed that 199,556 (39.3%) unigenes could be assigned into 58 functional categories in three main ontologies (Table S3). The top three largest GO terms in biological process were: 113,258 (21.7%) unigenes in cellular process (GO:0009987), 105,498 (20.2%) in metabolic process (GO:0008152), and 84,700 (16.2%) in single-organism process (GO:0044699). In total, 58,510 unigenes were annotated in 131 KEGG pathways using KOBAS v.2.0 software (Table S4). In the top three largest KEGG pathways, the most unigenes (2795, 4.8%) were annotated by plant-pathogen interactions (ko04626), followed by those for carbon metabolism (ko01200) (2741, 4.7%) and those for amino acid biosynthesis (ko01230) (2276, 3.9%). Other main pathways identified included starch and sucrose metabolism (ko00500), phenylpropanoid biosynthesis (ko00940), and plant hormone signal transduction (ko04075).

### 3.4. Identification and Functional Annotation of DEGs

**Figure 1:**
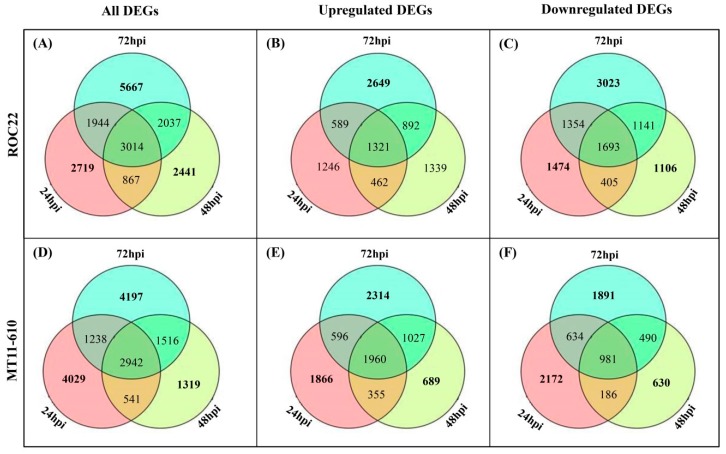
Venn diagram of all differentially expressed genes (DEGs) at 24–72 hpi defined by |log_2_(fold change)| > 1.5 and *p*-value < 0.005. All DEGs and up- and downregulated DEGs in (**A**–**C**) ROC22 (resistant to red stripe) and (**D**–**F**) MT11-610 (susceptible to red stripe) are shown.

A total of 29,887 DEGs were identified, of which 18,689 were present in the resistant cultivar ROC22 (R_Aaa vs R0_CK), and 15,782 were in the susceptible cultivar MT11-610 (S_Aaa vs S0_CK) (Figure 1). Furthermore, 8498 upregulated and 10,196 downregulated DEGs were found in ROC22, while MT11-610 had 8807 upregulated and 6984 downregulated DEGs. During the 24–72 hpi period, the number of regulated DEGs in ROC22 (2719) was lower than that for MT11-610 (4029) at 24 dpi, whereas the number of regulated DEGs in ROC22 (5667 and 2441) was higher compared to the number of DEGs in MT11-610 (4197 and 1319) at 48 dpi and 72 dpi, respectively (Figure 1). Notably, more upregulated DEGs were present in ROC22 at 48–72 hpi, compared with those for MT11-610 (Figure 1).

**Figure 2:**
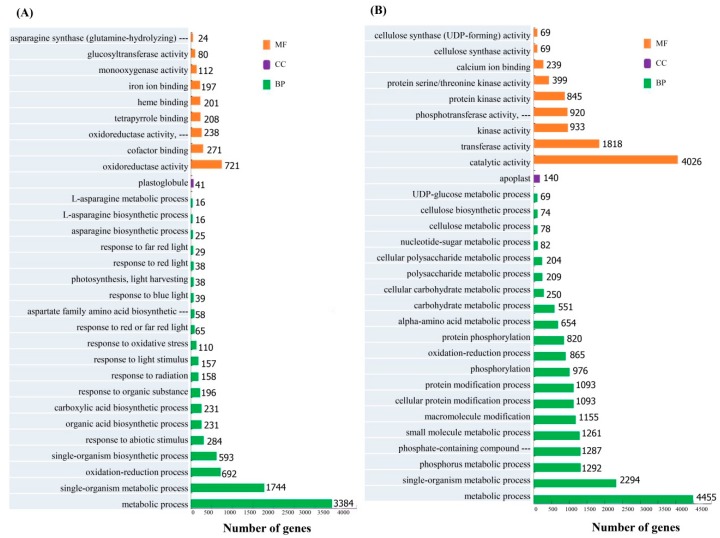
Gene Ontology (GO) annotation of differentially expressed genes (DEGs) in the *Aaa*-resistant cultivar ROC22. Up- (**A**) and downregulated (**B**) DEGs were annotated in three categories including biological process (BP), cellular component (CC), and molecular function (MF).

For the resistant cultivar ROC22, GO functional analysis revealed that 5464 and 6953 DEGs were up- and downregulated, respectively. Upregulated or downregulated DEGs were significantly enriched in two common GO categories, metabolic process and single-organism metabolic processes (Figure 2). The next most frequently enriched terms in upregulated and downregulated DEGs were oxidoreductase activity and catalytic activity, respectively. On the other hand, for the susceptible cultivar MT11-610, 5673 DEGs were upregulated and 4751 DEGs were downregulated. The top three GO categories for MT11-610 upregulated DEGs were respectively annotated as catalytic activity, single-organism metabolic process, and oxidation-reduction process (Figure S2A). For MT11-610 downregulated DEGs, the top three GO categories were respectively annotated as catalytic activity, carbohydrate metabolic process, and hydrolase activity (acting on glycosyl bonds) (Figure S2B).

KEGG analysis revealed that 3818 DEGs were significantly enriched in 67 pathways (Table S5). Of these pathways, 15 and 24 were present only in ROC22 and MT11-610, respectively, while 28 pathways occurred in both cultivars. The top ten KEGG pathways having the largest number of DEGs are listed in Table 2. Among these pathways, photosynthesis-antenna proteins and phenylpropanoid biosynthesis had 168 and 162 upregulated DEGs, respectively, for both cultivars. Meanwhile, 134 upregulated DEGs were enriched in ribosomes only in ROC22, and 102 upregulated DEGs were enriched in starch and sucrose metabolism only in MT11-610 (Table 2; Table S5). On the other hand, 186 downregulated DEGs were enriched in phenylpropanoid biosynthesis in both cultivars, whereas 145 and 80 downregulated DEGs involved in plant–pathogen interaction and plant hormone signal transduction, respectively, were seen only for ROC22, and 58 downregulated DEGs involved in starch and sucrose metabolism were seen only in MT11-610 (Table 2; Table S5).

### 3.5. Validation of DEGs by Real-Time qRT-PCR Analysis

**Figure 3:**
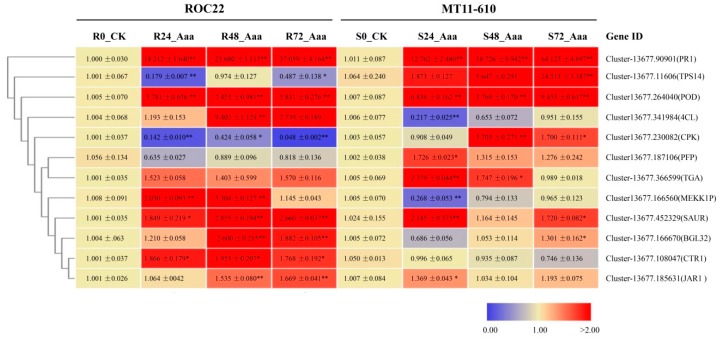
Heatmap depicting relative expression profiling of 12 candidate genes based on qRT-PCR (2^−ΔΔCT^) data. Samples were collected from ROC22 (resistant to red stripe) and MT11-610 (susceptible to red stripe) leaves at 0, 24, 48, and 72 h post-inoculation (hpi) with *Acidovorax avenae* subsp. *avenae* (*Aaa*). All data were normalized to the expression level of glyceraldehyde-3-phosphate dehydrogenase gene (*GAPDH*). Each column represents the mean ± standard error for three biological replicates. Significant changes in relative expression levels at each time point as compared to CK are marked with an asterisk (* *p* < 0.05; ** *p* < 0.01). Red and blue indicate up- and downregulation, respectively.

To validate the RNA-seq data, we randomly selected 12 DEGs for qRT-PCR analysis in both cultivars in response to *Aaa* infection. The 12 DEGs were involved in plant-pathogen interaction, plant hormone signal transduction, phenylpropanoid biosynthesis, monoterpenoid biosynthesis, and the pentose phosphate pathway. Transcriptional levels of two genes by RNA-seq (*SAUR* and *PR1*) were strongly upregulated in both cultivars, and seven genes (*MEKK1P*, *4CL*, *POD*, *BGL32*, *JAR1*, *CTR1*, and *TGA*) were highly expressed only in ROC22 (Table 3). Notably, the expression of two genes (*TPS14* and *PFP*) was highly upregulated in MT11-610 but depressed in ROC22. Overall, similar patterns of gene expression were observed between the qRT-PCR and RNA-seq-generated data. A significant positive correlation was observed between the two datasets, as indicated by a Pearson’s correlation coefficient r = 0.30438 (*p* = 0.0104; Figure 3).

### 3.6. DEGs in Photosynthesis and Carbon Metabolism Pathways

**Figure 4:**
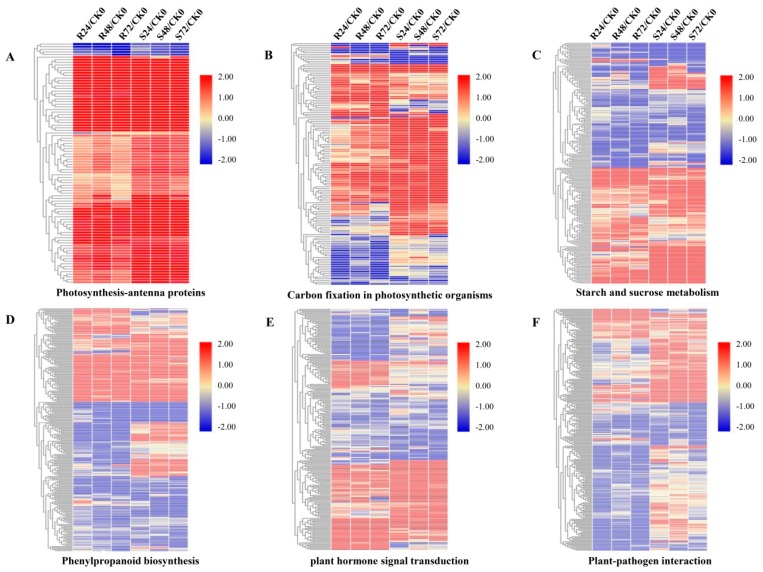
Hierarchical clustering of expression changes, log_2_(fold change), of differentially expressed genes (DEGs) involved in six main KEGG pathways in ROC22 (resistant to red stripe) and MT11-610 (susceptible to red stripe) at 0, 24, 48, and 72 h post-inoculation (hpi) with *Acidovorax avenae subsp. avenae* (*Aaa*). (**A**) photosynthesis-antenna proteins; (**B**) carbon fixation in photosynthetic organisms; (**C**) starch and sucrose metabolism; (**D**) phenylpropanoid biosynthesis pathway enriched by KEGG analysis; (**E**) plant hormone signal transduction and (**F**) plant–pathogen interaction pathways. R24/CK0, R24_Aaa vs. R0_CK;_R48/CK0, R48_Aaa vs. R0_CK; R72/CK0, R72_Aaa vs. R0_CK; S24/CK0, S24_Aaa vs. S0_CK;_S48/CK0, S48_Aaa vs. S0_CK; S72/CK0, S72_Aaa vs. S0_CK. Red and blue indicate up- and downregulation, respectively.

A number of DEGs related to photosynthesis and carbon metabolism pathways were differentially regulated in response to *Aaa* attacks (Figure S3; Table S6). Among these, 87 of 92 DEGs related to light-harvesting complex I chlorophyll a/b binding proteins 1-4 (LHCA1-4) and light-harvesting complex II chlorophyll a/b binding proteins 1-7 (LHCB1-7) in photosynthesis-antenna proteins (Ko00196) were significantly upregulated (Figure 4A; Table S6). Most DEGs enriched in carbon fixation in the photosynthetic organism pathway (ko00710) were significantly upregulated. However, only about half the DEGs for the carbon dioxide fixation (Calvin cycle) phase were upregulated, including DEGs for three key enzymes in this phase: ribulose-bisphosphate carboxylase small chain (rbcS, EC4.1.1.39), phosphoglycerate kinase (PGK, EC2.7.2.3), and glyceraldehyde 3-phosphate dehydrogenase (GAPDH, EC1.2.1.12). Three DEGs for phosphoribulokinase (PRK, EC2.7.1.19) were downregulated (Figure 4B; Table S6). Notably, the majority of DEGs (56/83) for the C_4_-dicarboxylic acid cycle were highly upregulated in both cultivars (Figure 4B; Table S6). These DEGs encoded key enzymes in this phase, such as phosphoenolpyruvate carboxylase (PEPC, EC4.1.1.31), malate dehydrogenase (oxaloacetate-decarboxylating) (NADP+) (maeB, EC1.1.1.40), and pyruvate orthophosphate dikinase (PPDK, EC2.7.9.1). Unexpectedly, five of seven DEGs encoding malate dehydrogenase (NADP+) (MDH, EC1.1.1.82), one of the key enzymes in the C_4_-dicarboxylic acid cycle, were significantly downregulated in both cultivars.

On the other hand, different genes involved in starch and sucrose metabolism pathways (ko00500) presented differential expression patterns (Figure 4C; Table S6). Of the key enzymes involved in sucrose metabolism, most DEGs related to sucrose synthase (SS, EC2.4.1.13), hexokinase (EC2.7.1.1), and fructokinase (EC2.7.1.4) were upregulated in both cultivars, whereas most DEGs related to sucrose-phosphate synthase (SPS, EC2.4.1.14) and beta-fructofuranosidase (sacA, EC3.2.1.26) were upregulated only in MT11-610. On the other hand, of four key genes involved in starch metabolism, the gene (Cluster-13677.257821) for UTP-glucose-1-phosphate uridylyltransferase (UGP2, EC2.7.7.9) was upregulated in MT11-610 but downregulated in ROC22. The gene for 1,4-alpha-glucan branching enzyme (glgB, EC2.4.1.18) was downregulated, particularly in MT11-610, which showed the upregulation of genes for starch synthase (glgA, EC2.4.1.21), whereas two genes encoding starch phosphorylase (glgP, EC2.4.1.1) were downregulated in both cultivars.

### 3.7. DEGs in Phenylpropanoid Biosynthesis

A total of 275 DEGs were involved in phenylpropanoid biosynthesis, another notable pathway in response of sugarcane to *Aaa* infection (Figure S4; Table S7). Of the 275 DEGs in this pathway, 85 and 117 DEGs were up- and downregulated, respectively, in both cultivars, while other DEGs were regulated depending on the cultivar genotype. Three key enzymes in phenylpropanoid biosynthesis were phenylalanine ammonia-lyase (PAL, EC4.3.1.24) or phenylalanine/tyrosine ammonia-lyase (PTAL, EC4.3.1.25), trans-cinnamate 4-monooxygenase [CYP73A, EC1.14.14.91 (formerly EC1.14.13.11)] and 4-coumarate-CoA ligase (4CL, EC6.2.1.12). Of 21 DEGs encoding PAL, six were downregulated in both cultivars; 15 genes were downregulated in ROC22 and 15 were upregulated in MT70-611. Three DEGs for PTAL were downregulated, whereas two DEGs for CYP73A were upregulated in the two cultivars. Of 12 DEGs encoding 4CL, five genes were upregulated, but the expression of three was depressed in both cultivars; another four genes were regulated according to cultivar genotype (Figure 4D; Table S7). Markedly significant upregulation of a gene (Cluster-13677.341984) encoding 4CL was verified by both qRT-PCR and RNA-seq (Figure 3; Table 3). About 44% (32/73) of DEGs encoding beta-glucosidase (EC3.2.1.21) that is associated with coumarin biosynthesis were increased in ROC22. Highly increased expression of a gene (Cluster-13677.166670) for beta-glucosidase (BGL32) was identified by both qRT-PCR and RNA-seq (Figure 3; Table 3). In addition, approximately 36.2% (38/105) of DEGs encoding peroxidase (EC1.11.1.7), the catalytic enzyme in final step of lignin biosynthesis, were upregulated in both cultivars, of which one gene (Cluster-13677.264040) was upregulated as indicated by qRT-PCR and RNA-seq assays (Figure 3; Table 3).

### 3.8. DEGs in Plant Hormone Signal Transduction Pathways

A total of 218 DEGs were significantly enriched in eight plant hormone signal transduction pathways, including those involving salicylic acid (SA), jasmonic acid (JA), ethylene (ET), abscisic acid (ABA), gibberellic acid (GA), cytokinin, auxin, and brassinosteroids (Figure S5; Table S8). Overall, about 36% (79/218) of DEGs involved in these pathways were upregulated, whereas expressions of 39% (84/218) of DEGs were depressed in both cultivars (Figure 4E; Table S8). In the SA signal transduction pathway, all five genes for the regulatory protein NPR1 (nonexpressor of pathogenesis-related genes 1) were downregulated and 4/10 genes for transcription factor TGA were upregulated in ROC22; Meanwhile, 8/9 genes for pathogenesis-related protein 1 (PR1) were upregulated in both cultivars. Our qRT-PCR data also revealed that the transcriptional level of a gene (Cluster-13677.366599) encoding TGA dramatically increased by 1.40–1.57-fold in ROC22 at 24–72 dpi (Figure 3). Additionally, the expression of a gene (Cluster-13677.90901) encoding PR1 was significantly increased by 14.2–37.1 and 12.8–64.1 folds in ROC22 and MT11-610, respectively (Figure 3). In the JA signal transduction pathway, one gene for jasmonic acid-amino synthetase (JAR1) was upregulated, but four genes for transcription factor (MYC2) were downregulated in both cultivars. In the ET signal transduction pathway, 20 DEGs were annotated and encoded six components, namely, ethylene receptor (ETR/ERS), serine/threonine-protein kinase (CTR1), ethylene-insensitive protein 2 (EIN2), ethylene-insensitive protein 3 (EIN3), EIN3-binding F-box protein (EBF1/2) and ethylene-responsive transcription factor 1 (ERF1). Five of seven genes for CTR1 were upregulated in both cultivars, of which the Cluster-13677.108047 gene was significantly induced with an increase of 3.70–4.90 and 1.78–1.96 folds in ROC22 based on RNA-seq and qRT-PCR data, respectively (Figure 3; Table 3). 

In the ABA signal transduction pathway, 47 DEGs were significantly enriched and involved in the ABA receptor PYR/PYL family (PYL) in addition to protein phosphatase 2C (PP2C), serine/threonine-protein kinase SRK2 (SnRK2), and ABA responsive element binding factor (ABF). Of these 47 DEGs, about 15 genes were upregulated, whereas 32 were downregulated, particularly PP2C and SnRK2, in ROC22. Similarly, most (13/18) DEGs were downregulated in ROC22 for the cytokinin signal transduction pathway, which includes *Arabidopsis* histidine kinase 2/3/4 (cytokinin receptor, CRE), histidine-containing phosphotransfer protein (AHP), two-component response regulator ARR-B family (ARR-B), and the two-component response regulator ARR-A family (ARR-A). In the auxin signal transduction pathway, 70 DEGs were significantly enriched in the LAX auxin influx carrier (AUX1 LAX family), auxin-responsive protein IAA (AUX/IAA), auxin response factor (ARF), auxin responsive GH3 gene family (GH3), and SAUR family protein (SAUR). Of these genes, 50% (35/70) were upregulated in ROC22, whereas 14 of 16 genes encoding GH3 were upregulated in both cultivars. Notably, the expression of Cluster-13677.452329 gene encoding SAUR was significantly enhanced by 4.34–5.26 and 1.85–2.86 folds, as evidenced by RNA-seq and qRT-PCR data, respectively (Figure 3; Table 3). In the GA signal transduction pathway, 18 DEGs were enriched. Of these, one gene encoding gibberellin receptor (GID1) and DELLA protein were downregulated, whereas one gene for phytochrome-interacting factor 3 (PIF3) and 14 genes for phytochrome-interacting factor 4 (PIF4) were highly expressed in both cultivars. In the brassinosteroid signal transduction pathway, most DEGs were downregulated in both cultivars, but especially in MT11-610.

### 3.9. DEGs in Plant-Pathogen Interaction Pathways

Overall, 235 DEGs were enriched in diverse plant-pathogen interaction pathways, including Ca^2+^-dependent signal transduction pathways and the mitogen-activated protein kinase (MAPK) signal transduction pathway, as well as those involved in activation of molecular chaperones (Figure S6; Table S9). Of these 235 DEGs, 61 were upregulated and 174 were downregulated in ROC22; conversely, 146 DEGs were highly expressed and 89 DEGs were depressed in MT11-610 (Figure 4F; Table S9). Expression of over 50% of genes for cyclic nucleotide gated channel (CNGC), eukaryotic calcium-binding protein (CML), calcium-dependent protein kinase (CPK), and WRKY transcription factor 33 (WRKY33) was depressed in ROC22, but these genes were highly expressed in MT11-610. Furthermore, qRT-PCR data verified that the transcriptional level of a gene (Cluster-13677.230082) encoding CPK was increased by 1.7- and 3.7-fold in MT70-6111 at 48 and 72 hpi, respectively, and was strongly decreased in ROC22 (Figure 3). In addition, 3/6 genes encoding mitogen-activated protein kinase kinase kinase 1, plant (MEKK1P) were significantly upregulated based on the RNA-seq data, while qRT-PCR data showed that transcripts of Cluster-13677.166560 gene for MEKK1P were enhanced by 1.1–3.3 folds at 24–72 hpi in two cultivars (Figure 3). Notably, 13 of 14 genes for molecular chaperone HtpG (HSP90A) were highly expressed, but 14 of 18 genes for respiratory burst oxidase (Rboh) were significantly depressed at 24–72 hpi in both cultivars.

## 4. Discussion

### 4.1. Overview of Gene Transcription in Sugarcane during Aaa Infection

Sugarcane is subject to a diverse range of causal pathogens including *Aaa* [11,12,13]. In this study, we performed a RNA-seq-based comparative transcriptome analysis to assess expression changes in sugarcane genes in response to *Aaa* infection. The RNA-seq data showed that 29,887 genes were differentially expressed upon *Aaa* infection and most changes appeared in the resistant cultivar ROC22, particularly within 72 hpi, suggesting that more defense-responsive genes were activated in this resistant cultivar relative to the susceptible cultivar MT11-610. A KEGG analysis showed that the majority of DEGs in sugarcane that responded to *Aaa* infection through transcriptional activation are involved in metabolic processes, single-organism metabolic processes, and oxidation-reduction processes in both resistant and susceptible cultivars. Induction of many genes involved in photosynthesis and carbon metabolism, plant-pathogen interaction, plant hormone signal transduction, and phenylpropanoid biosynthesis was also observed. These metabolic process and defense-responsive pathways were also previously reported to be involved in responses of sugarcane to infection by diverse pathogens, further suggesting that these responses are largely conserved in sugarcane exposed to biotic stress [25,26,35].

### 4.2. Regulation of Genes Related to Photosynthesis and Carbon Metabolism Pathways upon Aaa Infection

In the initial steps of photosynthesis, light energy is captured and converted into chemical energy and a large part of this light is absorbed by light-harvesting complexes (LHCs) that are peripherally associated with photosynthesis I (PSI) and photosynthesis II (PSII) [36,37]. In general, biotic stresses downregulate expression of genes related to photosynthesis [38]. Unexpectedly, our data revealed that genes involved in photosynthesis-antenna proteins (ko00196) were instead significantly upregulated, suggesting that expression of these proteins involved in the first step of photosynthesis may allow efficient light-driven electron transport to provide a defense strategy during early stages of *Aaa* infection. Meanwhile, a study by Santa Brigida et al. [26] demonstrated that expression of genes for ferredoxin [2Fe-2S] and ferredoxin-DNADP+ reductase, which are the final receptors for electrons in light-dependent reactions, were upregulated in response to *Aaa* attacks. Another study by Sun et al. [39] also showed that genes related to photosynthesis-antenna proteins are upregulated during infection of *Cucumis sativus* by *Cucurbit chlorotic yellows virus*.

On the other hand, we observed that approximately 50% of DEGs participating in carboxylation/Calvin-cycle reaction are upregulated, but genes encoding PRK (one of two characteristic enzymes in the Calvin cycle reaction) were downregulated, suggesting that *Aaa* infection represses carbon fixation since plant cells need more energy to fight against pathogen attacks or require defense mechanisms to overcome disease stress. Recently, Bi et al. reported that *N. benthamiana* plants with silencing of phosphoribulokinases (NbPRKs) were more resistant to *Rice stripe virus* (RSV), whereas transgenic plants overexpressing *NbPRK1* were more susceptible to RSV infection [40]. Furthermore, plant resistance was enhanced by silencing of either the small subunit of *N. benthamiana* rubisco (*NbRbCS*) or phosphoglycerate kinase (*NbPGK*) [40]. Except for DEGs encoding malate dehydrogenase (NADP+) (EC1.1.1.82), most DEGs related to PEPC (EC4.1.1.31), maeB (EC1.1.1.40) and PPDK (EC2.7.9.1) that play important roles in the C_4_-dicarboxylic acid cycle were upregulated in the C_4_ sugarcane crop, indicating that this crop may concentrate more CO_2_ in bundle-sheath cells via a mechanism involving a “CO_2_ pump” to promote the carboxylation reaction of rbcS and reduce photorespiration [41]. Notably, rbcS, PEPC, and PPDK are considered to be key enzymes for carbon fixation in sugarcane [42].

At the cellular level, sucrose is a key carbon source for plant growth, development, and defense [26,43]. Furthermore, sucrose is the main source of carbon and acts as an energy sink for sugarcane tissues [42,44]. SPS (EC3.1.3.24), SS (EC2.4.1.13) and invertase (INV, EC3.2.1.26) are key enzymes in sucrose metabolism [43]. Our findings showed that expression of genes encoding SS, hexokinase and fructokinase was upregulated, but genes encoding SPS and INV were downregulated, particularly in ROC22 (resistant to red stipe), suggesting that this resistant cultivar may accumulate more sucrose by reducing sucrose decomposition following *Aaa* infection. In contrast, most genes related to these key sucrose metabolism enzymes were upregulated in MT11-610 (susceptible to red stipe), indicating that this susceptible cultivar may accumulate more sucrose by enhancing sucrose synthesis after *Aaa* infection in order to meet energy demands of plant and/or bacterial growth. Indeed, pathogens often hijack host cells for a source of sugar [43,45]. Similarly, the invertase gene (*cwINV2*) was found to be upregulated in the SP70-1143 cultivar after inoculation with *Aaa* [26]. We also found that the expression of genes for starch synthase (EC2.4.1.21) was strongly upregulated, but genes for starch phosphorylase (EC2.4.1.1) were downregulated in both cultivars, suggesting that accumulation of starch in sugarcane has an important role during defense responses against *Aaa*. However, expression of starch synthase involved in starch biosynthesis was downregulated in leaves from SP70-1143 cultivar seedlings at 7 dpi with *Aaa* [26], which was in contrast to our findings in this study. This difference could be attributed to variations between these sugarcane cultivars. 

### 4.3. Regulation of Genes Involved in Phenylpropanoid Biosynthesis Pathways Exposed to Aaa Infection

A number of compounds containing a phenylalanine/tyrosine skeleton are directly or indirectly generated by the phenylpropanoid biosynthesis pathway, which plays various physiological functions in plants including phytoalexin-mediated defenses against herbivores and pathogens [46,47]. Of the first three enzymatic reactions with PAL, C4H (belonging to the CYP73A subfamily of the cytochrome P450-dependent monooxygenase superfamily) and 4CL, which is involved in the general phenylpropanoid pathway, are key enzymes that contribute to production of different phenylpropanoid precursors [47]. The expression of 4CL is altered in response to biotic stresses, reflecting its significant role in counteracting various biotic stresses [47]. Furthermore, peroxidase functions in the last step of lignin biosynthesis [48]. Lignin is an important structural component of the vascular secondary cell wall in plants, and provides not only mechanical strength and vascular integrity, but also plays important roles in conferring tolerance against abiotic and biotic stresses [49,50,51].

In this study we observed that expression of genes encoding PAL/PTAL was depressed in ROC22, but two genes for C4H as well as most genes for 4CL were upregulated in both resistant and susceptible cultivars. Furthermore, our RNA-seq and qRT-PCR data revealed that two genes (Cluster-13677.341984 and Cluster-13677.264040) encoding 4CL and peroxidase, respectively, were strongly upregulated in both cultivars. These findings suggested that increased expression of genes involved in lignin synthesis would be a common defense strategy employed by sugarcane in response to *Aaa* infection. A similar study by Su et al. revealed that expression of seven proteins (including 4CL) that are required in the lignin biosynthetic pathway was induced in sugarcane during *S. scitamineum* attack [35]. We also observed that transcriptional levels of a gene (Cluster-13677.166670) encoding beta-glucosidase were markedly increased based on both RNA-seq and qRT-PCR data, indicating that this gene may play a positive role in responses to *Aaa* attack. Notably, beta-glucosidase has broad functions in plants, such as recycling of cell-wall oligosaccharides, general defense, phytohormone signaling, secondary metabolism and scent release. This multi-functional role may be important for coordinating responses to diverse stresses [52].

### 4.4. Regulation of Genes in Plant Hormone Signal Transduction Pathways Play Important Roles in Response to Aaa Infection

Plant hormones play an essential role as signaling molecules in defense and immune responses by regulating hormone metabolic and signal transduction pathways, as well as through their ability to manipulate pathogen effectors [53]. Moreover, plant hormones act interdependently, through complex antagonistic or synergistic interactions [53,54]. Different hormone signaling pathways that activate and/or suppress defense during plant-pathogen interactions may function in sugarcane, as evidenced by different DEGs that play positive or negative roles in eight hormone signal transduction pathways. In this study, half of the DEGs in the eight hormone-related pathways were highly expressed in the resistant cultivar ROC22, but half were depressed in the susceptible cultivar MT11-610. Generally, SA and its derivative, methyl salicylate (MeSA), is typically identified as the signal(s) that are responsible for activating both local resistance and systemic acquired resistance (SAR) to biotrophic pathogens [53,55]. Our data showed that TGA and PR1 genes, two crucial components of SA signal transduction pathways, were highly expressed particularly in ROC22, suggesting these components can promote sugarcane resistance to *Aaa*.

Many studies have demonstrated that JA and methyl-jasmonate (MeJA) signaling molecules act as positive regulators of plant immunity against necrotrophic pathogens but are negative regulators in response to biotrophic pathogens [53,56,57]. We showed that a gene encoding JAR1 that enzymatically synthesizes (+)-7-iso-jasmonoyl-l-isoleucine (JA-Ile, an endogenous bioactive JA) by mediating isoleucine conjugation to JA was highly expressed upon *Aaa* infection, whereas expression of JA-regulated basic helix-loop-helix transcription factors (MYC2) was significantly depressed in both cultivars. These findings indicated that sugarcane infected by *Aaa* may display enhanced amounts of JA-Ile activity by promoting JAR1 activity and then depressing MYC2 activity, thereby inhibiting JA-dependent gene expression.

ET is a dual-function protein that is an important component of plant immune responses to pathogens [58]. CTR1 is a negative regulator of ET signaling [53] and its inhibition suppresses expression of downstream genes and transcription factors, thereby resulting in inactivation of ET-regulated genes [59]. In the present study, the levels of gene transcripts encoding CTR1 were strongly increased, whereas those for genes encoding EIN2, EIN3, and ERF1 were decreased in both cultivars, suggesting that the ET signal transduction pathway plays negative roles in response to *Aaa* infection. Also, Ntambo et al. revealed that this pathway was essential in sugarcane against *X. albilineans* [25]

For auxin signal transduction pathways, we found that expression of genes for GH3 and a gene (Cluster-13677.452329) for SAUR was significantly upregulated in sugarcane, similar to a previous study upon sugarcane in response to *X. albilineans* infection [25]. Plants infected successfully by biotrophic pathogens show significant increases in IAA concentration and thereby upregulation of GH3 [60,61]. Sugarcane exposed to *S. scitamineum* induced upregulation of auxin responsive proteins such as Aux/IAA, SAUR, and auxin-induced β-glucosidase [62]. Regarding GA hormone signal transduction pathway, it is important to note that two genes encoding DELLA and GID1 were highly expressed, whereas TF genes (PIF3 and PIF4) were downregulated, evidencing that degradation of DELLA proteins that act as negative regulators of GA responses during GA signaling ultimately relieves the negative regulation of the GA pathway to activate TFs that promote GA responses [63,64]. The dual roles of ABA in plants in response to resistance to biotrophic and necrotrophic pathogens depend on the environmental conditions during experiments [62]. Results in the present study suggest that the ABA signal transduction pathway plays an active role in sugarcane resistance to *Aaa*, as validated by the upregulation of PYR (a receptor complex of ABA) and downregulation of PP2C during *Aaa* infection. On the other hand, our data suggested that cytokinins involved in the regulation of plant defense responses against *Aaa* may depress expression of the components of this signal transduction pathway. Siemens et al. [65] reported that the expression of genes involved in cytokinin homeostasis (e.g., cytokinin synthases and cytokinin oxidases/dehydrogenases) was strongly downregulated in *Arabidopsis* during infection with *Plasmodiophora brassicae* and transgenic plants overexpressing cytokinin oxidase/dehydrogenase genes showed resistance against this pathogen. Collectively, plant hormones interdependently regulate complex signaling networks and are involved in crosstalk in plant immunity, thus an integrated understanding of phytohormone-mediated plant defense responses could inform the design of effective strategies for engineering crops that have disease resistance [62,63,66].

### 4.5. Regulation of Genes in Ca^2+^-Dependent and MAPK Signal Pathways in Response to Aaa Infection

Ca^2+^ is an important secondary messenger in signal transduction in plant cells [67]. In Ca^2+^ signaling, Ca^2+^ flux across membranes is promoted through the activity of several families of Ca^2+^-permeable channels, such as CNGCs. Plant cytoplasmic Ca^2+^ is sensed by different Ca^2+^ sensor proteins (e.g., CaMs, CMLs, CBLs, CDPKs), which further induce expression of downstream genes (e.g., transcription factors, NADPH oxidases genes) and mediate responses to abiotic and biotic stresses through reactive oxygen species (ROS) and/or nitrogen monoxide (NO) production [67,68]. CDPKs are involved not only in Ca^2+^- and ROS-mediated initiation of stress signaling, but also in hormone-regulated systemic signal propagation during pathogen infection [68,69]. On the other hand, the MAPK cascade generally has three components, a MAPK, a MAPKK (MAPK kinase) and a MAPKKK (MAPKK kinase), which play essential roles in plant growth and development as well as responses to abiotic and biotic stresses [67,68].

Our finding revealed that expression of most genes for CNGC, CML, CPK, and Rboh was particularly depressed in the resistant cultivar ROC22, whereas genes for MEKK1P (belongs to MAPKKK gene family) were highly expressed, suggested that sugarcane plants initiate defense responses during *Aaa* infection through genes induced by MAPK and the hypersensitive response (HR) mediated by HSP90, rather than through Ca^2+^ signaling, ROS or NO production. Indeed, Su et al. showed that Ca^2+^ signaling and ROS/NO pathways are not essential to smut resistance in sugarcane [35]. However, Santa Brigida et al. proposed that ROS production resulted from the strong induction of Rboh genes as part of an oxidative stress response against *Aaa* [26]. The different results concerning the role of ROS production in response to *Aaa* attack between these two independent studies could be attributed to differences in the environmental conditions used, such as different cultivars and sampling times. 

## 5. Conclusions

The present study demonstrated that sugarcane responds to *Aaa* infection through transcriptional activation of various genes, leading to significant changes in the levels of transcripts for genes involved in photosynthesis and carbon metabolism, phenylpropanoid biosynthesis, plant hormone signal transduction, and plant-pathogen interaction pathways. Although several gene responses and their related pathways were consistent with those reported in previous studies, our findings identified several important genes involved in the biosynthesis of lignin and coumarin, together with salicylic acid, jasmonic acid, ethylene and auxin signal transduction, and Ca^2+^-dependent as well as MAPK signal transduction pathways that had important positive roles in promoting the resistance of sugarcane to this pathogen. Our finding will provide a basis for the development of improved strategies to increase the resistance of modern sugarcane cultivars to *Aaa*.

## Figures and Tables

**Table 1:** Summary of sequencing data and de novo sequence assembly of sugarcane transcriptome under *Acidovorax avenae* subsp. *avenae* infection.

Characteristics of Transcripts/Unigenes	Number of Transcripts	Number of Unigenes
200–500 bp	278,011 (48.0%)	208,883 (41.2%)
500 bp–1 kbp	157,879 (27.2%)	155,399 (30.6%)
1–2 kbp	104,396 (18.0%)	104,024 (20.5%)
>2 kbp	39,275 (6.8%)	39,252 (7.7%)
Total number transcripts/unigenes	579,561 (100%)	507,558 (100%)
Minimal length (bp)	201	201
Median length (bp)	524	601
Maximal length (bp)	15,496	15,496
Mean length (bp)	789	860
N50 (bp) ^a^	1144	1200
N90 (bp) ^b^	344	395
Total number of nucleotides	457,476,577	436,705,780

^a^ The N50 value is defined as the contig length where half the assembly is represented by contigs of this size or longer. ^b^ N90 value is defined as the contig length where 90% of the assembly is represented by contigs of this size or longer.

**Table 2:** The top ten KEGG pathways having the largest number of differentially expressed genes (DEGs) in ROC22 (resistant to red stripe) and MT11-610 (susceptible to red stripe) sugarcane cultivars.

No.	Pathway ID	Pathway Name	Count (3818)	Percent (%)
1	ko00940	Phenylpropanoid biosynthesis	348	9.1
2	ko00196	Photosynthesis-antenna proteins	168	4.4
3	ko00500	Starch and sucrose metabolism	160	4.2
4	ko04075	Plant hormone signal transduction	153	4.0
5	ko04626	Plant-pathogen interaction	145	3.8
6	ko00520	Amino sugar and nucleotide sugar metabolism	140	3.7
7	ko03010	Ribosome	134	3.5
8	ko00460	Cyanoamino acid metabolism	131	3.4
9	ko00710	Carbon fixation in photosynthetic organisms	124	3.2
10	ko00250	Alanine, aspartate and glutamate metabolism	115	3.0

**Table 3:** Transcriptional expression of 12 randomly selected differentially expressed genes (DEGs) in ROC22 (resistant to red stripe) and MT11-610 (susceptible to red stripe) sugarcane cultivars based on RNA-seq data.

No.	Gene ID	Gene Annotation ^b^	KEGG Orthology	ROC22 ^a^			MT11610 ^a^		
				24 hpi	48 hpi	72 hpi	24 hpi	48 hpi	72 hpi
1	Cluster-13677.166560	MEKK1P	K13414(Plant-pathogen interaction)	2.74	3.47	3.07	−0.07	0.13	0.26
2	Cluster-13677.90901	PR1	K13449(Plant hormone signal transduction)	5.13	5.85	6.53	2.72	2.47	2.86
3	Cluster-13677.341984	4CL	K01904(Phenylpropanoid biosynthesis)	1.94	2.85	2.22	−0.52	0.56	−0.26
4	Cluster-13677.264040	POD	K00430(Phenylpropanoid biosynthesis)	3.34	3.04	2.48	0.79	0.88	0.74
5	Cluster-13677.166670	BGL32	K01188(Phenylpropanoid biosynthesis)	1.97	2.60	2.75	0.03	0.39	1.20
6	Cluster-13677.452329	SAUR	K14488(Plant hormone signal transduction)	4.34	4.76	5.26	1.01	7.45	3.39
7	Cluster-13677.185631	JAR1	K14506(Plant hormone signal transduction)	1.60	1.74	1.76	0.51	0.83	0.89
8	Cluster-13677.108047	CTR1	K14510(Plant hormone signal transduction)	3.70	4.90	4.44	−0.41	0.85	−0.34
9	Cluster-13677.366599	TGA	K14431 (Plant hormone signal transduction)	6.75	6.38	8.01	0.27	0.39	0.04
10	Cluster-13677.230082	CPK	K13412(Plant-pathogen interaction)	−7.103	−4.81	−7.87	−0.02	1.44	−1.55
11	Cluster-13677.11606	TPS14	K15086(Monoterpenoid biosynthesis)	0.12	−1.21	−0.32	4.63	6.92	7.09
12	Cluster-13677.187106	PFP	K00895(Pentose phosphate pathway)	−1.64	−4.01	−2.44	7.47	7.61	7.89

^a^ The transcriptional expression levels of RNA-seq shown as log_2_(fold change). ^b^ MEKK1P, mitogen-activated protein kinase kinase kinase 1, plant; PR1, pathogenesis-related protein 1; 4CL, 4-coumarate-CoA ligase; POD(EC1.11.1.7), peroxidase; BGL32(EC3.2.1.21), beta-glucosidase; SAUR, SAUR family protein; JAR1, jasmonic acid-amino synthetase; CTR1, serine/threonine-protein kinase CTR1; TGA, transcription factor TGA; CPK, calcium-dependent protein kinase; TPS14, (3S)-linalool synthase; PFP(EC2.7.1.90), Pyrophosphate: fructose-6-phosphate 1-phosphotransferase (PFP).

## Supplementary Materials

The following are available online at https://www.mdpi.com/2076-2607/8/1/10/s1, Figure S1: Summary of annotation from public databases (A) and species distribution of the top BLAST hits (B) for assembled high-quality unigenes. NR, NCBI non-redundant protein database; Nt, NCBI nucleotide sequences; KO, KEGG (Kyoto Encyclopedia of Genes and Genome) Ortholog; SwissProt, a manually annotated and reviewed protein sequence database; Pfam, protein family; KOG, euKaryotic Ortholog Groups; GO, Gene Ontology; Figure S2: Gene Ontology (GO) annotation of differentially expressed genes (DEGs) in the Aaa-susceptible cultivar MT11-610. Up- (A) and downregulated (B) DEGs were annotated in three categories: biological process (BP), cellular component (CC), and molecular function (MF); Figure S3: Schematic diagram of relationships between differentially expressed genes (DEGs) enriched in photosynthesis and carbon metabolism pathways as identified by KEGG analysis. All genes encoding catalytic enzymes revealed by RNA-sequencing data are highlighted by a yellow box; Figure S4: Schematic diagram of relationships between differentially expressed genes (DEGs) involved in the phenylpropanoid biosynthesis pathway as identified by KEGG analysis. All genes encoding genes/transcription factors revealed by RNA-sequencing data are highlighted by a gray box and those genes that were also validated by qRT-PCR are highlighted by a green box; Figure S5: Schematic diagram of relationships between differentially expressed genes (DEGs) enriched in plant hormone signal transduction pathways as identified by KEGG analysis. All genes encoding genes/transcription factors revealed by RNA-sequencing data are highlighted by a gray box and those genes that were also validated by qRT-PCR are highlighted by a green box. Those that showed no change by either RNA-sequencing or qRT-PCR assay are enclosed by white boxes; Figure S6: Schematic diagram of relationships between differentially expressed genes (DEGs) enriched in plant-pathogen interaction pathways as identified by KEGG analysis. All genes encoding genes/transcription factors revealed by RNA-sequencing data are highlighted by a gray box and those genes that were also validated by qRT-PCR are highlighted by a green box. Those that showed no change by either RNA-sequencing or qRT-PCR assay are enclosed by white boxes; Table S1: Primers used for qRT-PCR of the 12 randomly selected differentially expressed genes (DEGs); Table S2: Overview of RNA-sequencing reads derived from 24 libraries for *Aaa* (*Acidovorax avenae* subsp. *avenae*)- and mock-infected samples from resistant (ROC22) and susceptible (MT11-610) cultivars; Table S3: Gene Ontology (GO) annotation of all unigenes identified in the two sugarcane cultivars, ROC22 and MT11-610; Table S4: Kyoto Encyclopedia of Genes and Genomes (KEGG) annotation of all unigenes identified in the two sugarcane cultivars, ROC22 and MT11-610; Table S5: Differentially expressed genes (DEGs) enriched in each KEGG pathway in resistant (ROC22) and susceptible (MT11-610) sugarcane cultivars; Table S6: Transcript levels (log_2_Fold Change) determined by RNA sequencing for differentially expressed genes (DEGs) in ROC22 and MT11-610 enriched in three KEGG pathways: photosynthesis-antenna proteins (ko00196), carbon fixation in photosynthetic organisms (ko00710), and starch and sucrose metabolism (ko00500); Table S7: RNA sequencing results for transcript levels (log_2_Fold Change) of differentially expressed genes (DEGs) enriched in the KEGG phenylpropanoid biosynthesis pathway (ko00940); Table S8: RNA sequencing results for transcript levels (log_2_Fold Change) for differentially expressed genes (DEGs) in ROC22 and MT11-610 enriched in the KEGG plant hormone signal transduction pathway (ko04075); Table S9: RNA sequencing results for transcript levels (log_2_Fold Change) of differentially expressed genes (DEGs) in ROC22 and MT11-610 enriched in the KEGG plant-pathogen interaction pathway (ko04626).
