# Supplementary material for: Global Gene Responses of Resistant and Susceptible Sugarcane Cultivars to Acidovorax avenae subsp. avenae Identified Using Comparative Transcriptome Analysis
Source: Microorganisms. 2019 Dec 19;8(1):10. doi: 10.3390/microorganisms8010010 (PMC7022508; doi:10.3390/microorganisms8010010)
Supplement: Supplementary file 1 [file microorganisms-08-00010-s001.zip › Figure S1-20191213.pdf]

(A)

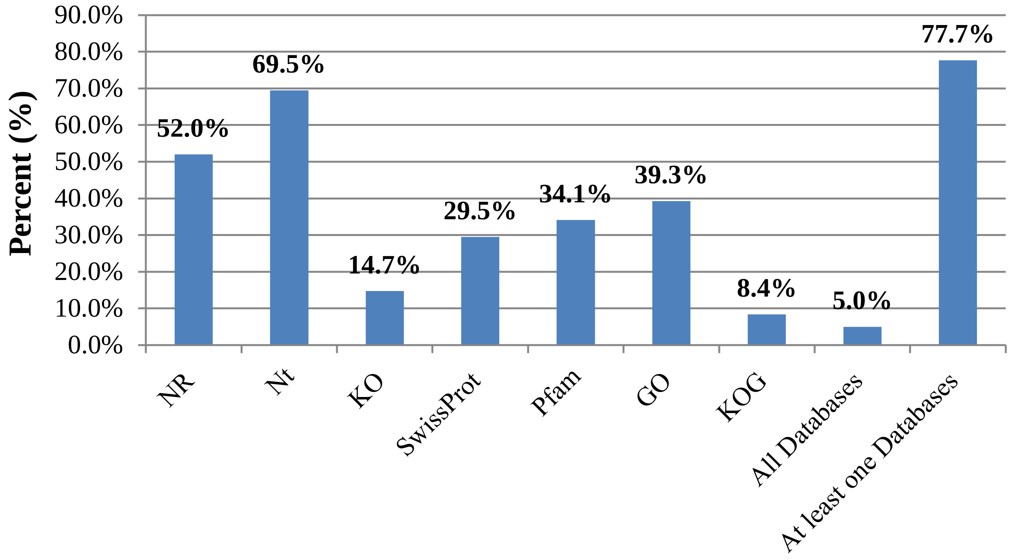

(B)

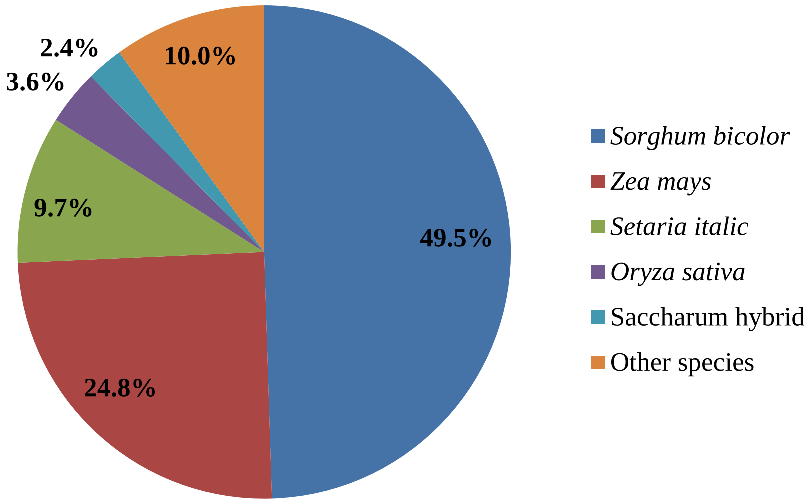

Figure S1. Summary of annotation from public databases (A) and species distribution of the top BLAST hits (B) for assembled high-quality unigenes. NR, NCBI non-redundant protein database; Nt, NCBI nucleotide sequences; KO, KEGG (Kyoto Encyclopedia of Genes and Genome) Ortholog; SwissProt, a manually annotated and reviewed protein sequence database; Pfam, protein family; KOG, euKaryotic Ortholog Groups; GO, Gene Ontology.
