# Supplementary material for: Global Gene Responses of Resistant and Susceptible Sugarcane Cultivars to Acidovorax avenae subsp. avenae Identified Using Comparative Transcriptome Analysis
Source: Microorganisms. 2019 Dec 19;8(1):10. doi: 10.3390/microorganisms8010010 (PMC7022508; doi:10.3390/microorganisms8010010)
Supplement: Supplementary file 1 [file microorganisms-08-00010-s001.zip › Figure S2-20191213.pdf]

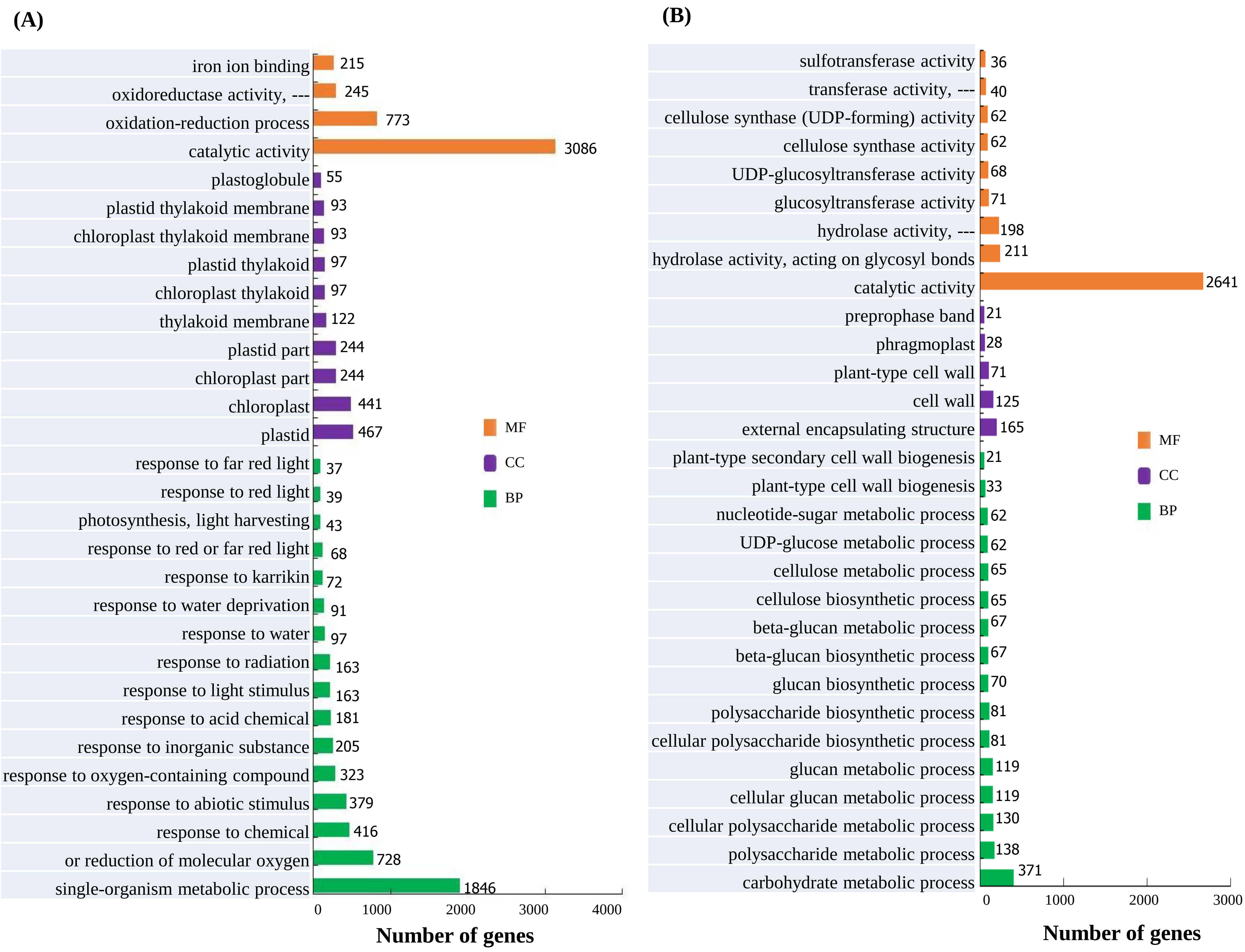

Figure S2. Gene Ontology (GO) annotation of differentially expressed genes (DEGs) in the *Aaa*-susceptible cultivar MT11-610. Up- (A) and downregulated (B) DEGs were annotated in three categories: biological process (BP), cellular component (CC), and molecular function (MF).
