# Supplementary material for: Global Gene Responses of Resistant and Susceptible Sugarcane Cultivars to Acidovorax avenae subsp. avenae Identified Using Comparative Transcriptome Analysis
Source: Microorganisms. 2019 Dec 19;8(1):10. doi: 10.3390/microorganisms8010010 (PMC7022508; doi:10.3390/microorganisms8010010)
Supplement: Supplementary file 1 [file microorganisms-08-00010-s001.zip › Figure S3-20191213.pdf]

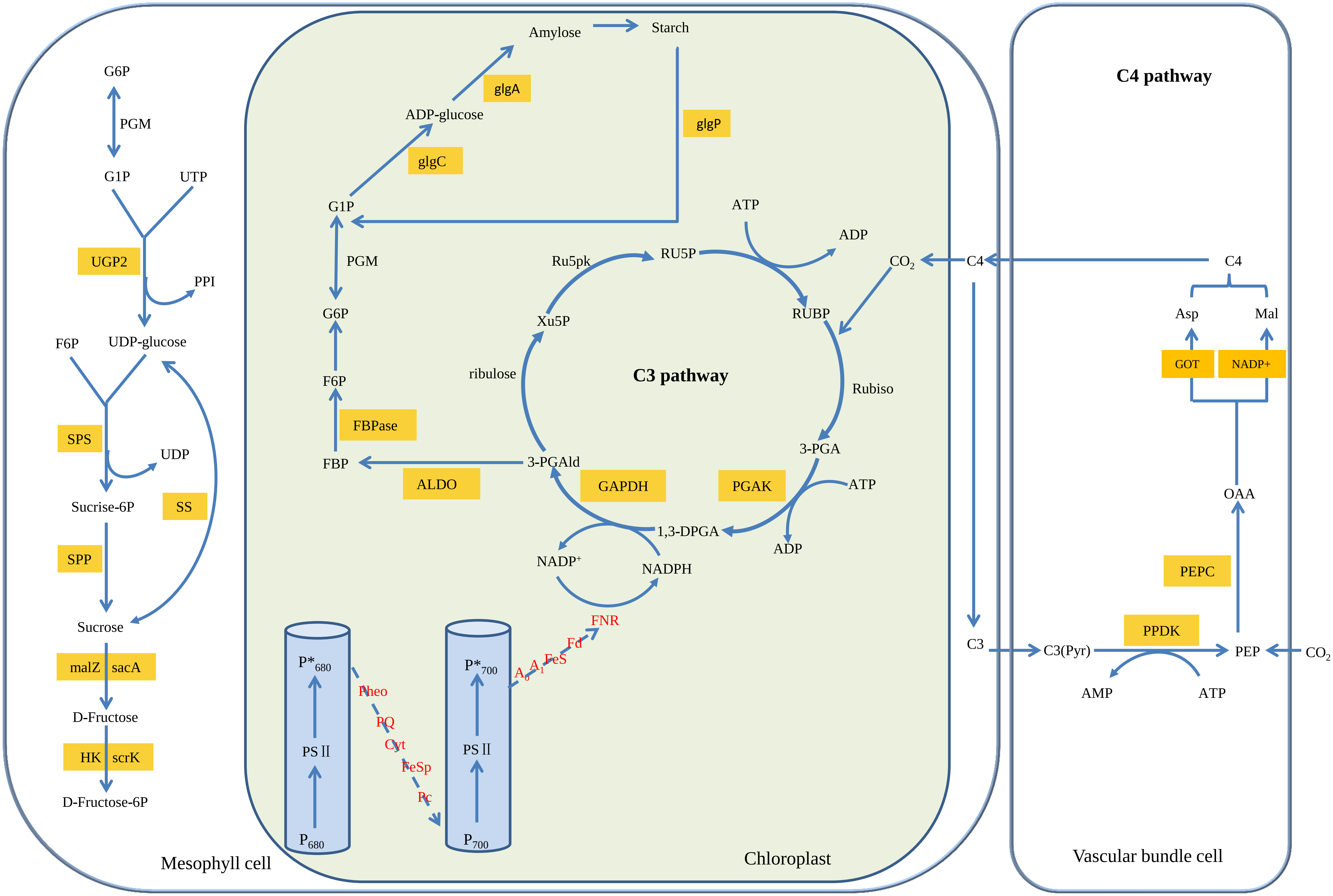

Figure S3. Schematic diagram of relationships between differentially expressed genes (DEGs) enriched in photosynthesis and carbon metabolism pathways as identified by KEGG analysis. All genes encoding catalytic enzymes revealed by RNA-sequencing data are highlighted by a yellow box.
