# Supplementary material for: Global Gene Responses of Resistant and Susceptible Sugarcane Cultivars to Acidovorax avenae subsp. avenae Identified Using Comparative Transcriptome Analysis
Source: Microorganisms. 2019 Dec 19;8(1):10. doi: 10.3390/microorganisms8010010 (PMC7022508; doi:10.3390/microorganisms8010010)
Supplement: Supplementary file 1 [file microorganisms-08-00010-s001.zip › Figure S4-20191213.pdf]

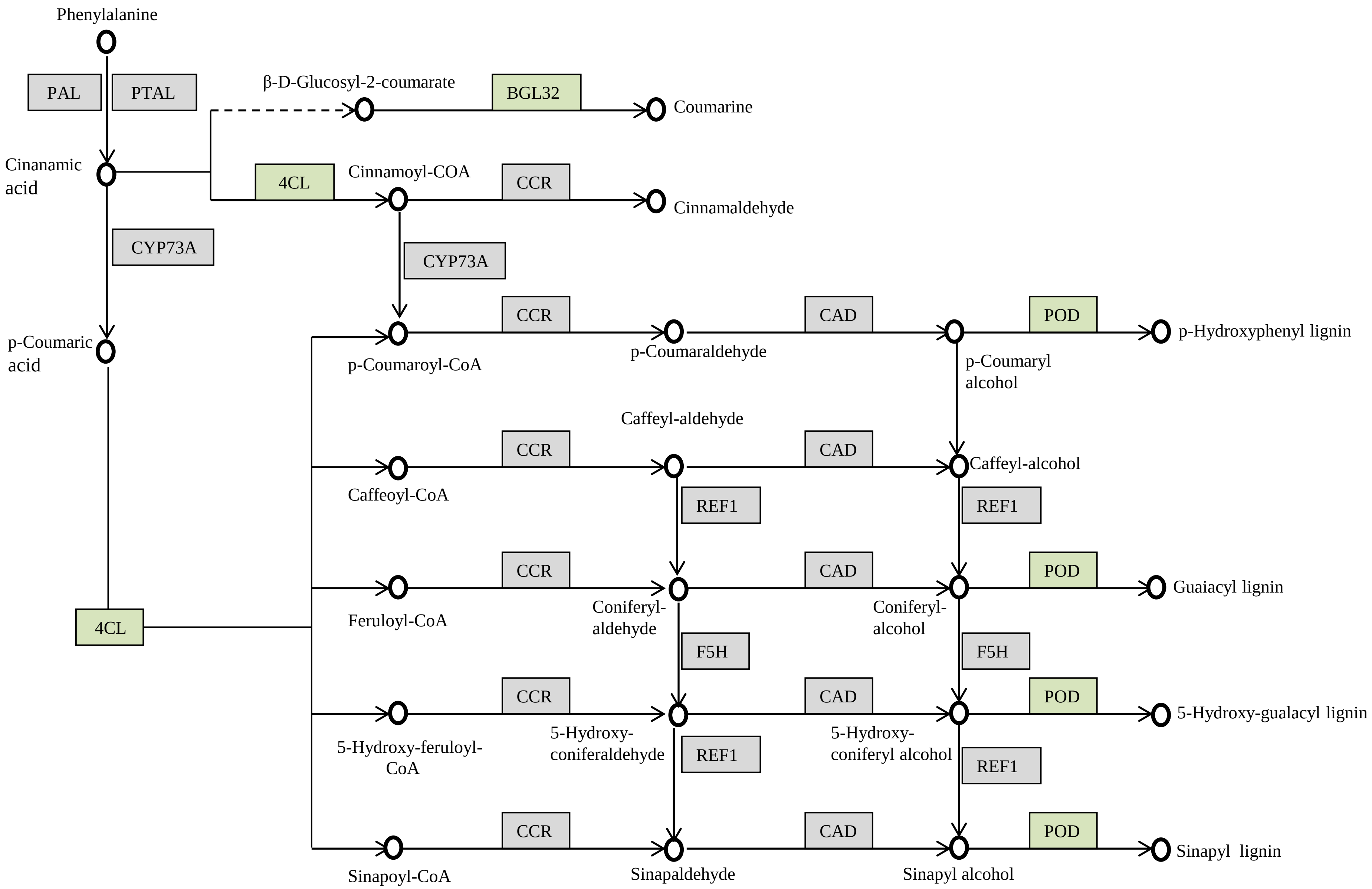

Figure S4. Schematic diagram of relationships between differentially expressed genes (DEGs) involved in the phenylpropanoid biosynthesis pathway as identified by KEGG analysis. All genes encoding genes/transcription factors revealed by RNA-sequencing data are highlighted by a gray box and those genes that were also validated by qRT-PCR are highlighted by a green box.
