# Supplementary material for: Global Gene Responses of Resistant and Susceptible Sugarcane Cultivars to Acidovorax avenae subsp. avenae Identified Using Comparative Transcriptome Analysis
Source: Microorganisms. 2019 Dec 19;8(1):10. doi: 10.3390/microorganisms8010010 (PMC7022508; doi:10.3390/microorganisms8010010)
Supplement: Supplementary file 1 [file microorganisms-08-00010-s001.zip › Figure S5-20191213.pdf]

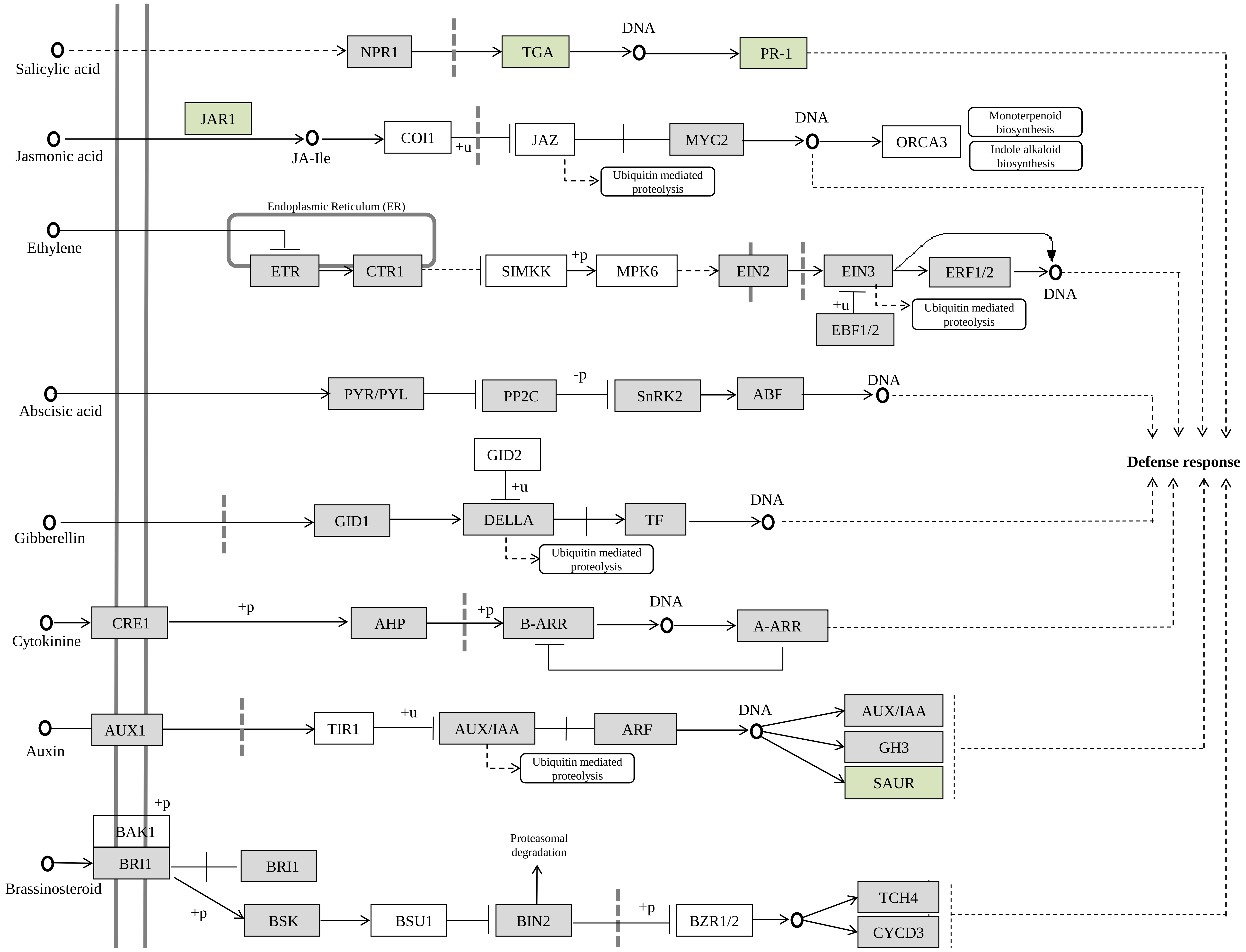

Figure S5. Schematic diagram of relationships between differentially expressed genes (DEGs) enriched in plant hormone signal transduction pathways as identified by KEGG analysis. All genes encoding genes/transcription factors revealed by RNA-sequencing data are highlighted by a gray box and those genes that were also validated by qRT-PCR are highlighted by a green box. Those that showed no change by either RNA-sequencing or qRT-PCR assay are enclosed by white boxes.
