# Supplementary material for: Global Gene Responses of Resistant and Susceptible Sugarcane Cultivars to Acidovorax avenae subsp. avenae Identified Using Comparative Transcriptome Analysis
Source: Microorganisms. 2019 Dec 19;8(1):10. doi: 10.3390/microorganisms8010010 (PMC7022508; doi:10.3390/microorganisms8010010)
Supplement: Supplementary file 1 [file microorganisms-08-00010-s001.zip › Table S1-20191213.pdf]

Table S1. Primers used for qRT-PCR of the 12 randomly selected differentially expressed genes (DEGs).

Gene ID	Gene description (EC number) [a]	Forward primer (5'→3')	Reverse primer (5'→3')	Tm (°C) [b]	Amplicon size (bp)	Efficiency [c]
Cluster-13677.166560	MEKK1P	CCACGGAGGCCATGTTCATC	GCTTCCCAGAAGAGCACCAC	60	86	0.99
Cluster-13677.90901	PR1	GACTGCCAGCTGGTTCACTC	AACTGCTTCTCGGACACCCA	59	116	0.92
Cluster-13677.341984	4CL	GCGACGTCGTCCTTCTCCTC	GGCGGTGTAGAGTGGGTTGG	62	110	0.99
Cluster-13677.264040	POD(EC1.11.1.7)	CGGTGAATACCACGGTGAAC	ACAAACTGCTCGAAGAAGGC	56	181	0.91
Cluster-13677.166670	BGL32(EC3.2.1.21)	ATGGTTCATGCATCCGTTGG	CCGGAATCCTTCTTCTCGGA	56	183	0.96
Cluster-13677.452329	SAUR	CCGATTCTGCCATCGAGGTG	TTTGTGGACACGACGGAAGC	58	145	0.99
Cluster-13677.185631	JAR1	CAGAGCTCCGGTTCATCTGC	AGCTTTGCTGCCTCCTCAAC	58	103	0.94
Cluster-13677.108047	CTR1	AACCTTTCAATTGTGACTGA	ATTGGAGGATTACGCTTATG	50	158	0.99
Cluster-13677.366599	TGA	TAATTTACGGCTGCAAACAC	CCAAAGAGAACTCAAAGCAC	52	121	1.02
Cluster-13677.230082	CPK	ATCTGCGTGGGTTAGTTCAC	ATCTGACTCAGGACCAGACC	56	200	1.02
Cluster-13677.11606	TPS14	TGCATGACATGTCTCACCTG	TCAGACTCAGGTGGTAAGGG	56	159	0.97
Cluster-13677.187106	PFP(EC2.7.1.90)	CACCAGGAATCAGATAACTC	GAACTTCCTTCCCTTGTATC	53	123	0.98
	GAPDH (reference gene)	CACGGCCACTGGA AGCA	TCCTCAGGGTTCCTGATGCC	58	101	1.01

[a] MEKK1P, mitogen-activated protein kinase kinase kinase 1, plant; PR1, pathogenesis-related protein 1; 4CL, 4-coumarate-CoA ligase; POD, peroxidase; BGL32, beta-glucosidase-32; SAUR, small auxin up RNA/SAUR-like auxin-responsive protein family; JAR1, jasmonic acid-amido synthetase; CTR1, serine/threonine-protein kinase CTR1; TGA, transcription factor TGA; CPK, calcium-dependent protein kinase; TPS14, S-linalool synthase; PFP, pyrophosphate--fructose-6-phosphate 1-phosphotransferase; GAPDH, glyceraldehyde-3-phosphate dehydrogenase. [b] Tm, melting temperature of primers. [c] SYBR green based qRT-PCR efficiencies were calculated according to the standard curves of serial dilutions of total cDNA from the sugarcane leaf sample.
