# Supplementary material for: Global Gene Responses of Resistant and Susceptible Sugarcane Cultivars to Acidovorax avenae subsp. avenae Identified Using Comparative Transcriptome Analysis
Source: Microorganisms. 2019 Dec 19;8(1):10. doi: 10.3390/microorganisms8010010 (PMC7022508; doi:10.3390/microorganisms8010010)
Supplement: Supplementary file 1 [file microorganisms-08-00010-s001.zip › Table S2-20191120.pdf]

Table S2. Overview of RNA-sequencing reads derived from 24 libraries for *Aaa* (*Acidovorax avenae subsp. avenae*)- and mock-infected samples from resistant (ROC22) and susceptible (MT11-610) cultivars [a]

	ROC22				MT11-610			
Description	R0_CK	R24_Aaa	R48_Aaa	R72_Aaa	S0_CK	S24_Aaa	S48_Aaa	S72_Aaa
Raw reads	60,922,564	62,218,354	55,408,650	58,752,069	58,224,389	65,758,625	59,100,537	62,717,477
Clean reads	59,959,395	61,259,067	54,282,803	57,434,943	57,284,497	64,485,665	57,875,395	61,269,024
Total mapped	38,662,587	40,040,937	35,805,262	38,037,384	37,291,365	42,863,503	38,245,766	41,022,658
Mapped (%)	65.1	65.5	66.4	66.7	65.4	66.5	66.3	67.3
Clean bases (G)	8.99	9.19	8.14	8.62	8.59	9.67	8.68	9.19
Q30 (%)	92.5	92.3	92.9	92.6	91.0	92.4	92.7	93.0
GC Content (%)	55.2	55.7	56.6	55.9	56.0	57.7	57.2	57.5

[a] The data from the average of three replicates in each treatment.
